# Multivariate analysis of morphology, behaviour, growth and developmental timing in hybrids brings new insights into the divergence of sympatric Arctic charr morphs

**DOI:** 10.1186/s12862-021-01904-8

**Published:** 2021-09-07

**Authors:** Quentin J.-B. Horta-Lacueva, Sigurður S. Snorrason, Michael B. Morrissey, Camille A.-L. Leblanc, Kalina H. Kapralova

**Affiliations:** 1 Institute of Life and Environmental Sciences, University of Iceland, Askja - Náttúrufræðihús, Sturlugötu 7, 102 Reykjavík, Iceland; 2 School of Biology, University of St Andrews, Sir Harold Mitchell Building, Greenside Place, St Andrews, UK; 3 Department of Aquaculture and Fish Biology, Hólar University, Háeyri 1, 550 Sauðárkrókur, Iceland

**Keywords:** Adaptive divergence, Ecological speciation, Development, Trait covariance, Sympatry, Resource polymorphism, Hybridization

## Abstract

**Background:**

Studying the development of fitness related traits in hybrids from populations diverging in sympatry is a fundamental approach to understand the processes of speciation. However, such traits are often affected by covariance structures that complicate the comprehension of these processes, especially because the interactive relationships between traits of different nature (e.g. morphology, behaviour, life-history) remain largely unknown in this context. In a common garden setup, we conducted an extensive examination of a large suit of traits putatively involved in the divergence of two morphs of Arctic charr (*Salvelinus alpinus*), and investigated the consequences of potential patterns of trait covariance on the phenotype of their hybrids. These traits were measured along ontogeny and involved growth, yolk sac resorption, developmental timing (hatching and the onset of exogeneous feeding), head morphology and feeding behaviour.

**Results:**

Growth trajectories provided the strongest signal of phenotypic divergence between the two charr. Strikingly, the first-generation hybrids did not show intermediate nor delayed growth but were similar to the smallest morph, suggesting parental biases in the inheritance of growth patterns. However, we did not observe extensive multivariate trait differences between the two morphs and their hybrids. Growth was linked to head morphology (suggesting that morphological variations in early juveniles relate to simple allometric effects) but this was the only strong signal of covariance observed between all the measured traits. Furthermore, we did not report evidence for differences in overall phenotypic variance between morphs, nor for enhanced phenotypic variability in their hybrids.

**Conclusion:**

Our study shed light on the multivariate aspect of development in a context of adaptive divergence. The lack of evidence for the integration of most traits into a single covariance structure suggested that phenotypic constraints may not always favour nor impede divergence toward ecological niches differing in numerous physical and ecological variables, as observed in the respective habitats of the two charr. Likewise, the role of hybridization as a disruptive agent of trait covariance may not necessarily be significant in the evolution of populations undergoing resource polymorphism.

**Supplementary Information:**

The online version contains supplementary material available at 10.1186/s12862-021-01904-8.

## Background

Understanding how phenotypic traits subjected to divergent selection evolve is essential to comprehend the processes of adaptive divergence and speciation [1–4]. In this context, reproductive isolation often relates to reduced fitness in hybrids whose values for specific traits under divergent selection are intermediate or fall outside of the range of parental values (i.e. transgressive characters) [5–7]. However, traits are rarely independent entities because of functional trade-offs [8, 9], developmental constraints [10], genetic constraints like pleiotropy and linkage disequilibrium [10, 11] or the effect of correlational selection [12, 13]. Furthermore, traits belonging to various processes (i.e. life-history, development, behaviour) and encompassing different ontogenetic stages are often intertwined (for examples in fish and amphibians, see [14–23]. While these evolutionary aspects have long been studied in the field of quantitative genetics, and while classical models of ecological speciation are based on the effects of pleiotropy and/or of large sets of co-selected genes [2, 24, 25], little is known about the importance of covarying traits in a context of speciation [2, 6], especially regarding the development of the hybrid phenotypes. Studies on hybridisation often focus on one or a limited number of traits, most often related to morphology and to some extent to physiology and behaviour [2] (but see [26], for a thorough study on life-history and morphology), which reveals the need for multivariate, longitudinal studies on the ontogeny of hybrids.

Characterizing the development of first-generation hybrids (F_1_ hybrids) in a multivariate framework would be a first significant step to understand the effects of trait covariance in speciation. Additive mechanisms generating intermediate mean trait values in F_1_ hybrids are expected to be fairly common [1, 27–29]. However, recent theoretical and empirical studies report evidence for dominance in individual traits often causing parent bias (i.e. hybrids having closer trait values to one parent rather than being intermediate [30, 31] or showing extreme phenotypes [32–37]). In addition to mean trait values, increased phenotypic variance in F_1_ hybrids is expected, presumably because of new allelic combinations and epistatic effects [27]. Likewise, trait covariance and correlations should be strengthened in many cases [27], but hybridization is also expected to relax trait correlations [38]. Finally, independent traits affected by parent-bias are likely to generate “trait mismatches” that might be detrimental in the wild [30]. Given the high number of traits potentially involved in divergence processes and the importance of trait covariance, it becomes critical to thoroughly study the development of F_1_ hybrids in a multivariate context before studying the evolutionary consequences (e.g. selection against hybrids as a reproductive barrier).

Polymorphic fish from Northern freshwater lakes are particularly well-suited models to study the processes of phenotypic divergence [39]. The evolution of these fish fits the narrative of resource polymorphism, through which different forms (i.e. morphs) have emerged from ancestral populations that invaded multiple, unoccupied niches within the same geographical system [40]. Such diversification often follows the colonisation of deglaciated lakes, where the diverging morphs (generally segregating between benthic and pelagic habitats) differ in morphology, life-history traits and/or behaviour [41, 42]. Various levels of reproductive isolation are encountered among these systems, ranging from single populations with continuous variation, to discrete varieties with more-or-less reversible reproductive barriers, to completely reproductively isolated species [5, 43, 44]. In recent years a growing number of cases have been reported where post-glacial morphs are found (at least in their current state) in sympatry [44–46]. These geographical and evolutionary systems facilitate the explorations of the mechanisms of adaptive divergence and speciation because of the reduced confounding effects of long and complex evolutionary histories [47].

Using multivariate phenotypic data on morphology, behaviour and ontogeny, and considering different developmental stages, we characterized phenotypic variations among two of the four sympatric morphs of Arctic charr (*Salvelinus alpinus*) from Thingvallavatn, Iceland, and of their hybrids. These morphs are the small-benthic (SB) and the planktivorous charr (PL), which constitute two genetically differentiated populations [48–50] and differ in head and body shape, habitat use, diet, life-history and parasites [51–53]. The SB charr live in the interstitial spaces of a lava matrix forming the stony littoral zone of the lake, where they forage on benthic invertebrates. The PL charr utilize the pelagic zone of the lake where they feed on zooplankton and emerging chironomids. Because these two habitats differ extensively in their physical and ecological characteristics [53, 54], the different selective regimes experienced by each morph are expected to affect a wide variety of traits. Previous studies already indicate that the PL and the SB charr have evolved genetically based differences in their embryonic growth [52], craniofacial development [55, 56], and foraging strategy [57]. The two morphs overlap in their spawning time and places [58] but recent estimates of gene flow indicate substantial reproductive isolation [49, 50]. Fertile hybrids (at least of the generation F_1_) can however easily be produced in laboratory. In the wild, selection against hybrids is therefore likely to be an important reproductive barrier between these two morphs.

Using a common garden set-up, we reared the offspring of SB, PL charr and their hybrids, keeping track of individuals from hatching until about 3 months after the onset of exogeneous feeding. We assessed traits related to morphology and development (hatching date, initial size and growth, yolk sac size and resorption, developmental trajectory of the head shape). These measurements enabled us not only to test for differences in average value, variances and covariances of traits between types of crosses, but also to assess whether and how these traits covary with other traits measured later in life, and which were related to morphology (shape of the feeding apparatus), behaviour (feeding intensity) and growth after the onset of exogeneous feeding (Table 1). We first hypothesised that the two morphs have rapidly diverged in every aspect of their developmental phenotype. If the two morphs have evolved towards distinct multivariate fitness optima, we expected to observe (1) differences between pure-morph offspring in average trait values. Because divergence may affect already covarying traits or involve correlational selection, we also expected (2) differences in trait variances and covariances to be established between the two pure-morph offspring.

**Table 1:** Variables selected for generating the phenotypic (**P**) variance–covariance matrices (one per cross type)

Category	Variable name
Development	Standard length at hatching (D1)
	Standard length at the onset of first feeding (D3)
	Growth from hatching (D1) to 20 days post-hatching (D2)
	Growth from 3–4 weeks after the onset of exogeneous feeding (D3) to 9–11 weeks after the onset of exogeneous feeding (D4)
	Yolk sac size at hatching (D1)
	Yolk sac conversion
Behaviour	Latency to start feeding at the start of observational trials

Our second hypothesis was that hybrids show a unique ontogenetic phenotype composed of characters with various inheritance patterns (additive, dominant, over dominant). These characters would provide cues regarding the potential of reproductive isolation and/or hybridization to generate phenotypic variation.

## Results

### Developmental deficiencies

We first investigated whether higher mortality or higher occurrence of heavy malformations in hybrids can be observed in our common garden study. The proportion of individuals dying after hatching or killed because of heavy malformations appeared to be higher in the SB × SB offspring and the hybrids than in the PL × PL offspring (PL × PL: 0.03; SB × SB: 0.32; hybrids: 0.29). However, after implementing a Generalized Mixed models (GLMM) with family (i.e. the egg clutch) as a random effect, these differences only appear as trends (posterior modes [95% CrIs] of the survival probability on the latent scale = PL × PL: 3.24 [1.80; 5.83], SB × SB: 0.62 [− 0.60; 1.92], hybrids: 0.91 [− 0.05; 1.80], family effect: 0.02 [0.00; 2.42]).

### Differences at the level of individual traits

We collected multivariate longitudinal individual-based data on ontogeny (standard length, yolk sac resorption, growth before and after the onset of exogeneous feeding, timing of the onset of exogeneous feeding), trophic morphology (head shape) and feeding behaviour (feeding activity and feeding performance). With the exception of shape data, differences in average trait value and in variances were estimated by fitting GLMMs and by making inferences based on the overlap between 95% High Posterior Credible intervals (95% CrI).

**Fig. 1:**
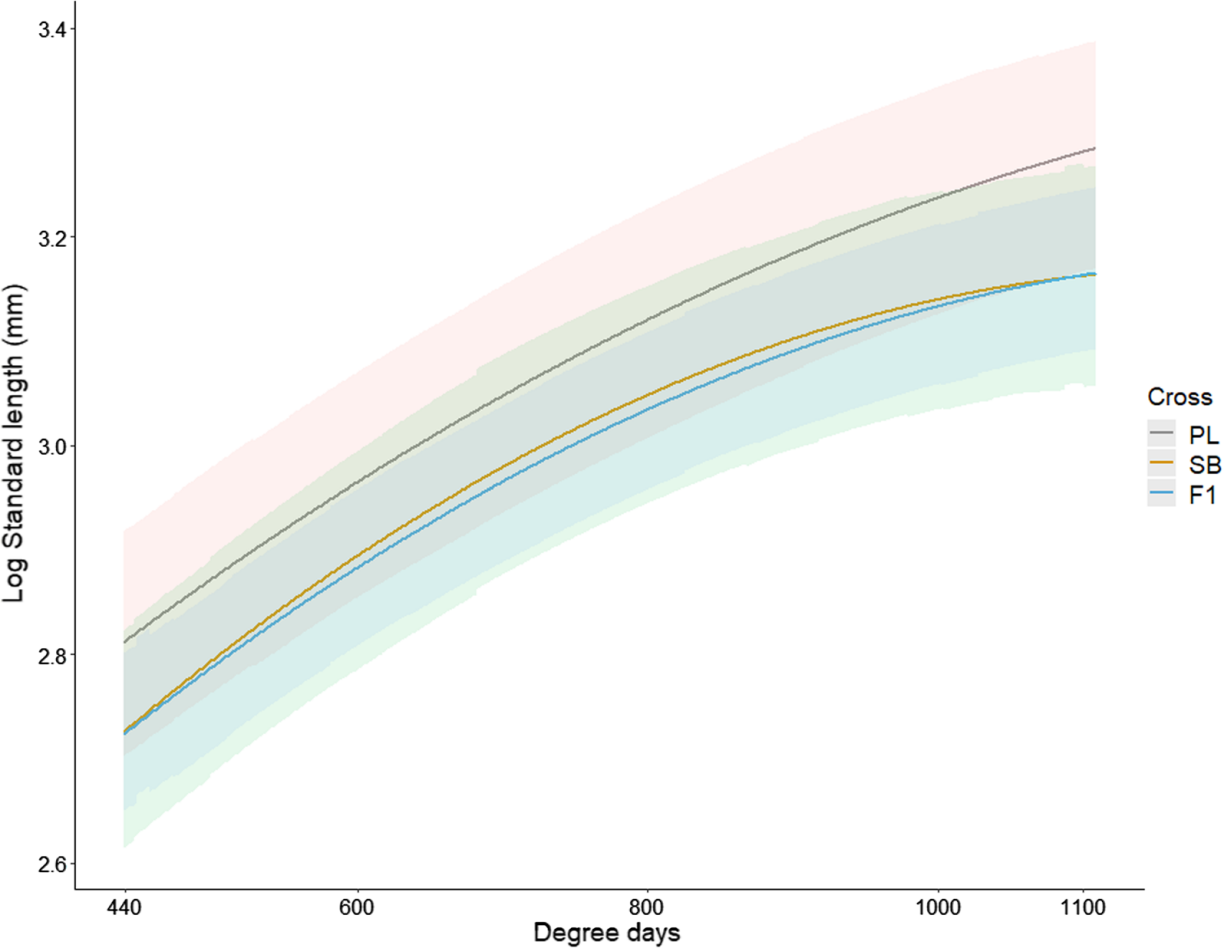
Growth trajectories of each cross type. The growth period under study started at hatching (ca. 400 °C d) and ended 3 months after the onset of exogeneous feeding (ca. 1100 °C d). PL: PL × PL offspring, SB: SB × SB offspring, F_1_: first-generation hybrids. Lines: predicted values, bands: 95% confidence intervals

Longitudinal size measurements (standard length) indicated that the SB × SB and the hybrids differed from the PL × PL offspring in their growth trajectories (Fig. 1). We observed a trend for lower intercepts in the SB × SB offspring and the hybrids than in PL × PL offspring (posterior modes [95% CrIs] of log_10_(standard length) = PL × PL: 3.09 [2.97; 3.19], SB × SB: 2.98 [2.90; 3.11], hybrids: 2.98 [2.92; 3.07]). Furthermore, lower slopes and small second order polynomial terms were observed in the SB × SB offspring and the hybrids compared to the PL × PL offspring (slopes = PL × PL: 6.14 [5.89; 6.38], SB × SB: 5.77 [5.38, 5.95], hybrids: 5.73 [5.55; 5.92]; second order polynomial terms = PL × PL: − 0.70 [− 0.85; − 0.55], SB × SB = − 1.14 [− 1.37; − 0.99], hybrids: − 1.00 [− 1.11; − 0.85]). These results indicate a slower and a more decelerating growth in the SB × SB offspring and the hybrids than in the PL × PL offspring.

Using Geometric morphometric data from photographs of the embryos, we did not observe strong differences between the types of crosses in mean yolk sac area at hatching nor in the rate of yolk sac resorption (Table 2). The hybrids and the SB × SB offspring appeared to have smaller yolk sac sizes at hatching and the hybrids tended to have faster resorption rate, although wide overlaps in 95% intervals confer low levels of certainty to these patterns.

**Table 2:** Posterior estimates of the fixed effects from the Multi-response Generalized Linear Mixed Effect Model on the yolk sac area (mm^2^)

	Posterior mode	95% CrI
Response (yolk area at D1)	2.65	1.86; 3.65
Response (yolk area at D2)	2.63	1.56; 3.36
Yolk area at D1 × log(standard length at D1)	0.11	0.06; 0.15
Yolk area at D2 × log(standard length at D2)	0.11	0.06; 0.15
Yolk area at D1 × Cross type SB × SB	− 0.15	− 1.38; 1.17
Yolk area at D2 × Cross type SB × SB	− 0.03	− 1.25; 1.29
Yolk area at D1 × Cross type F_1_ hybrids	− 0.21	− 1.21; 0.93
Yolk area at D2 × Cross type F_1_ hybrids	− 0.16	− 1.16; 0.99

The PL × PL cross type is the base line. D1: hatching, D2: 20 days post-hatching. See Additional file 1: Table S5 for the details of the model

**Fig. 2:**
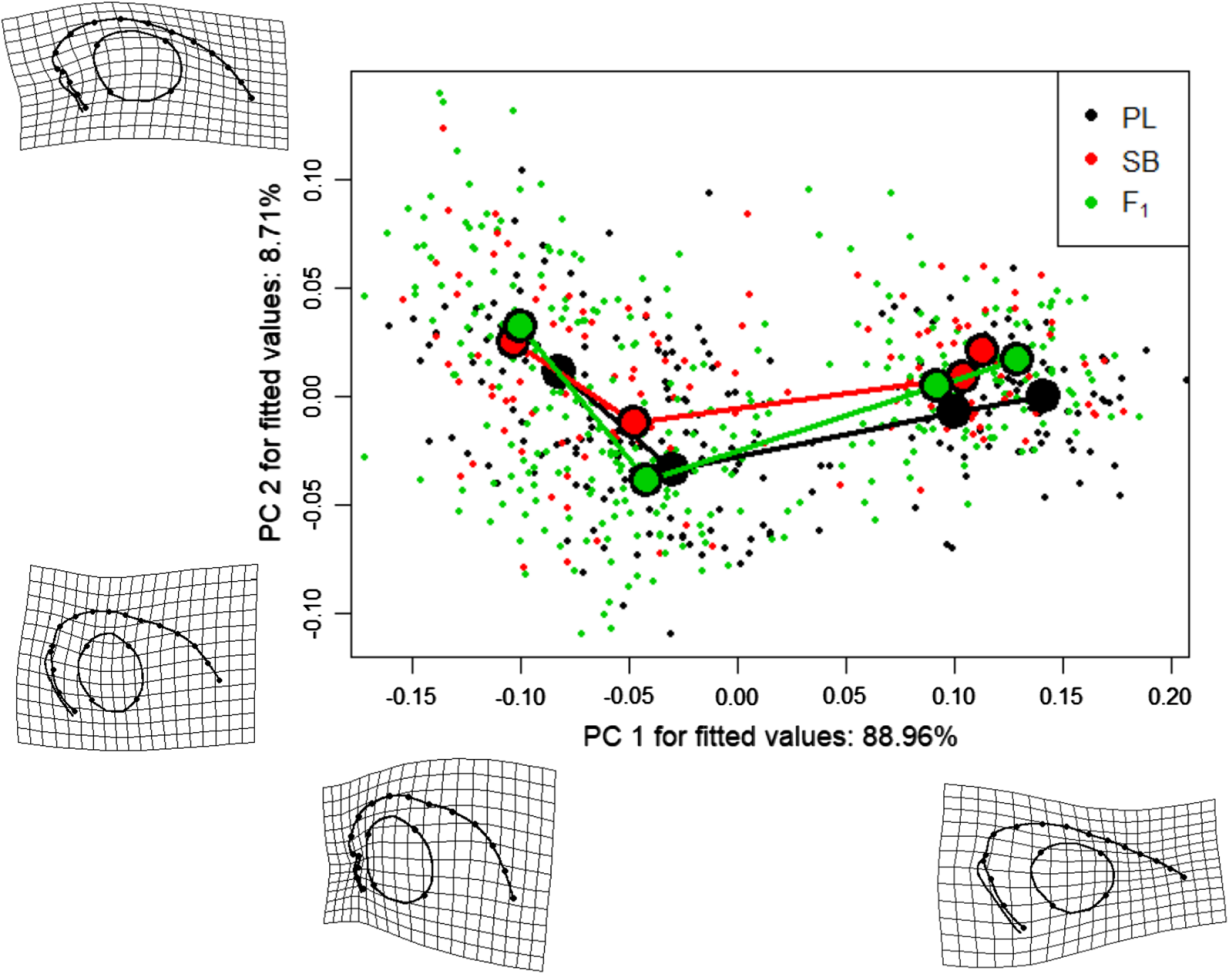
Ontogenetic trajectories and deformation grids at the extremes of each Principal Component axis. The shapes are not corrected for size. SB = SB × SB offspring, PL = PL × PL offspring, F_1_ = firs-generation hybrids. The circles are the mean shapes of each cross type at a given developmental time point (from left to right: hatching, 20 days post hatching, onset of exogeneous feeding, 3 months after the onset of exogeneous feeding)

Head shape variation between cross types was estimated with Analyses of the Procrustes residuals (Randomized Residuals Permutation Procedure) of Geometric Morphometric data from the same set of photographs used for the yolk sac analyses. These analyses indicated that size was related to most of the variation among specimens while no effect of the cross type in itself was observed (Table 3). The ontogenetic trajectories of the head shape did not differ significantly between the types of crosses (Fig. 2, Additional file 1: Table S1). No differences between types of crosses in the variances of the head shapes were observed from the disparity analyses at hatching and at the onset of exogeneous feeding (absolute differences in Procrustes variances < 0001, *p*-value > 0.1 in all the pairwise comparisons).

**Table 3:** Formula and results of the regression on Procrustes residuals of the head shapes in the specimen reared individually

Formula					
Procrustes coordinates ~ log_10_(size) + Cross/Family + log_10_(size) × Cross/Family + Age × Coss/Family					
Table of variance					
Effects	*d.f*	*SS*	*R*^*2*^	*Z*	*p*
Log(size)	1	5.17	0.62	7.80	< 0.01
Cross type	2	0.06	0.01	− 1.18	0.88
Age	3	0.63	0.07	9.47	< 0.01
Cross type × Family	5	0.25	0.03	8.07	< 0.01
Cross type × log(size)	2	0.02	0.00	− 2.64	1.00
Cross type × Age	6	0.07	0.01	− 3.09	1.00
Cross type × log(size) × Family	5	0.06	0.01	4.08	< 0.01
Cross type × Age × Family	15	0.10	0.01	3.63	< 0.01
Residuals	626	2.06	0.24	–	–
Total	665	8.40	–	–	–

Families are nested within cross type. Age: Sampling time point, Size: Centroid size

**Fig. 3:**
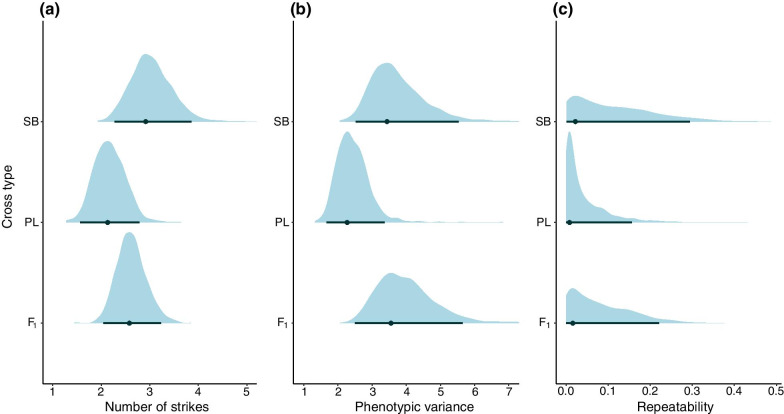
Number of food items attacked by the focal fish during the feeding trials. **a** Number of attacks, **b** total variance, and **c** Repeatability (R) as a measure of consistent between-individual differences. Categories: SB = SB × SB offspring, PL = PL × PL offspring, F_1_ = hybrids. Blue shapes: Posterior densities, dots: Posterior modes, bars: and 95% CrIs

Finally, we estimated the date of the onset of exogenous feeding of each individual through daily observations and studied variations in feeding behaviour among cross types (3–4 weeks after the onset of exogeneous feeding) by conducing three sessions of behavioural observations per individual (focal sampling [59]). We did not observe differences among cross types either in the mean or in the variances of the date of the onset of exogeneous feeding, the estimated dates being very close to one another (largest posterior mode difference = 5 days, Additional file 1: Table S2). There was no apparent difference between groups in the propensity to start feeding during the experimental trials on feeding behaviour (PL × PL = 0.76 [0.57; 0.87]; SB × SB = 0.70 [0.53; 0.88]; hybrids = 0.65 [0.51; 0.77]; posterior mode [95% CrI], observed scale). However, the PL × PL offspring showed a higher level of consistent individual differences (repeatability) in their propensity to start feeding (R = 0.41 [0.23; 0.53], posterior mode [95% CrI]) than the SB × SB offspring (R = 0.00 [0.00; 0.25]) and the hybrids (R = 0.00 [0.00; 0.27]). The estimated number of captured food items also appeared slightly lower in PL × PL offspring than in the SB × SB conspecifics, although no strong inference can be made in light of the overlapping 95% CrI (Fig. 3a). PL × PL individuals also tended to show lower variance than the SB × SB individuals and the hybrids in the number of attacked items (Fig. 3b). The comparison of these estimates indicated that the three types of crosses showed low to null levels of consistent differences between individuals in this feeding behaviour (Fig. 3c). We did not observe differences between cross types in the latency to start feeding (all differences in posterior mode > 1 s.; all 95‰ CrI highly similar, Additional file 1: Table S3) nor in the propensity to use the bottom of the container, the water column or the surface of the water when foraging (Additional file 1: Figure S1a–i).

### Trait covariance structure and correlations (head shape excluded)

We studied the patterns of trait covariance (excluding head shape) by generating a phenotypic matrix of variance–covariance (**P** matrix) for each cross type. We first compared the cross types on the basis of each component of **P** (trait variances and trait correlations), then on the general properties of **P** (matrix size, eccentricity and angle), and finally by assessing through Krzanowski’s common subspaces method [60] whether parts of **P** (i.e. particular suits of covarying traits) differed in variance.

Within **P**, trait variance and correlation structures of each cross type revealed higher variance in growth after exogeneous feeding in the SB × SB offspring than in the PL × PL offspring (Table 4). More dissimilarity was observed in the hybrids, which were associated with reduced variances in size and growth (from hatching to the onset of first feeding) compared to the two pure-morph offspring. We did not report evidences for differences in trait correlations between cross types (Table 4).

**Table 4:** Posterior modes and 80% CrIs credible intervals (CrIs) of trait variance that showed nonoverlaps in CrIs between at least two cross types (all CrIs of trait correlations overlapped)

Trait	PL × PL	SB × SB	F_1_ hybrids
Standard length (D1)*	0.14 [0.11; 0.18]	0.20 [0.15; 0.27]	0.08 [0.07; 0.10]
Standard length (D3)^†^	0.21 [0.14; 0.26]	0.21 [0.15; 0.28]	0.10 [0.08; 0.13]
Growth from D1 to D3^†^	0.18 [0.15; 0.24]	0.22 [0.18; 0.33]	0.13 [0.11; 0.16]
Growth from D3 to D4^†^	0.31 [0.23; 0.42]	0.68 [0.52; 1.12]	0.42 [0.32; 0.57]
Yolksac-relative area (D1)	0.15 [0.11; 0.18]	0.22 [0.15; 0.27]	0.11 [0.09; 0.14]
Yolksac-conversion	0.15 [0.12; 0.18]	0.21 [0.16; 0.29]	0.12 [0.10; 0.15]

D1 = hatching, D2 = 20 days post hatching, D3 = onset of exogeneous feeding, D4 = 3 months after the onset of exogeneous feeding

^*^95% CrI also nonoverlapping

^†^90% CrI also nonoverlapping

**Fig. 4:**
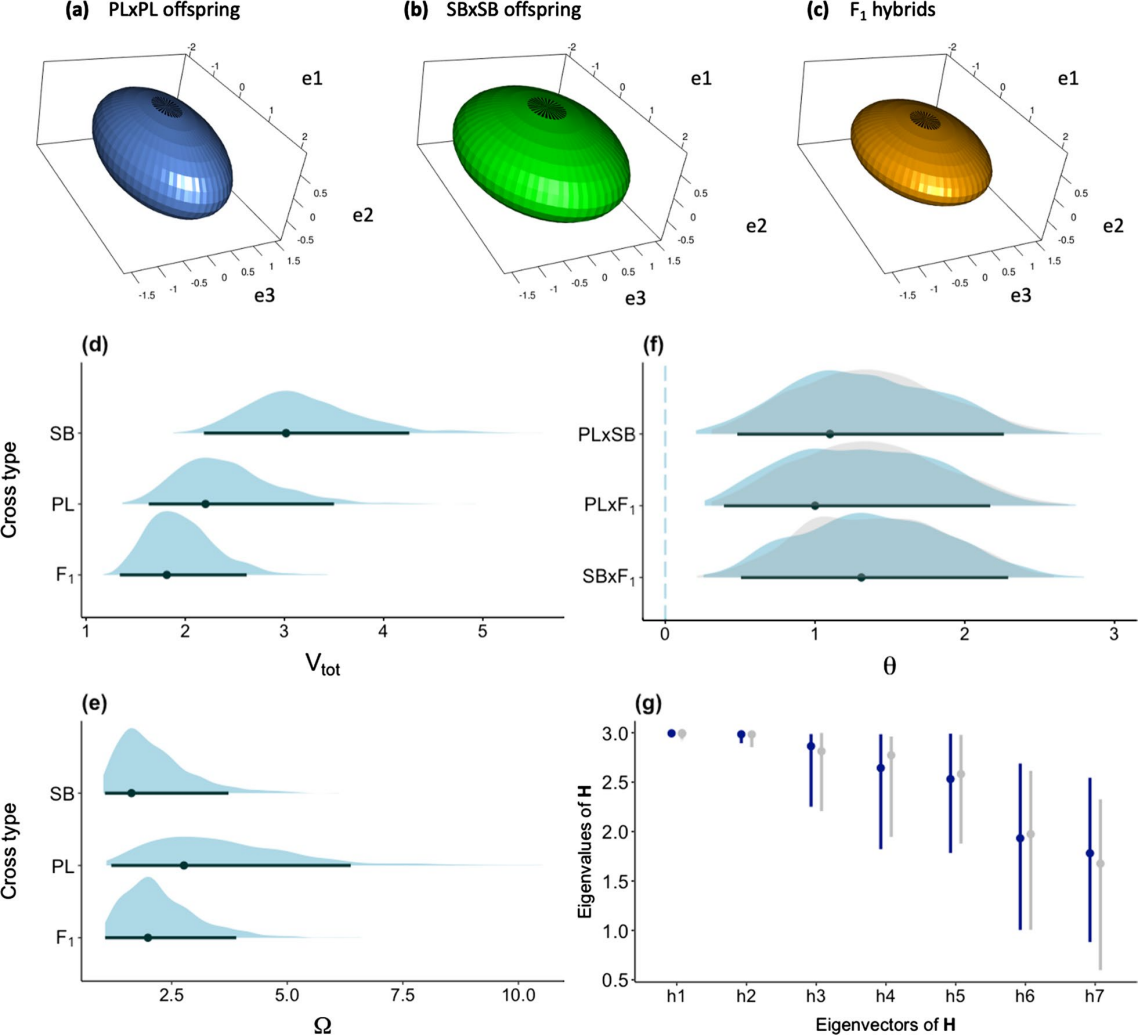
Summary properties the **P** matrices of each cross type. **a**–**c** Ellipsoid representations of the posterior modes of each matrix projected onto a subspace defined by the first three eigenvectors of **P** from the PL × PL cross. The axes explain 80%, 69% and 77% of the variance of **P** in the PL × PL, the SB × SB and the hybrid crosses, respectively. **d**–**f** Posterior densities, posterior modes and 95% CrIs of the three summary estimates of the matrices of phenotypic variance of each cross type, being **d** the overall phenotypic variance (*V*_*tot*_), **e** the eccentricity (*Ω*), and **f** the angle (*θ*) between the first eigenvectors. Densities of the angle estimates between **P** of a cross type (from top to bottom: PL × PL, PL × PL, SB × SB) and a random matrix are shown in light grey. Cross type: SB = SB × SB offspring, PL = PL × PL offspring, F_1_ = hybrids. **g** Posterior modes and 95% CrIs of **H** for the observed data (dark blue) and the randomized P matrices (light grey)

The differences in variance also appeared as trends at the scale of **P** matrices, V_tot_ tending to be the largest in the SB × SB offspring and the smallest in the hybrids (Fig. 4a–c). This indicates higher overall phenotypic variation in the SB × SB than in the PL × PL and the hybrids. A trend for more phenotypic constraints (more eccentricity) also appeared in the PL × PL offspring. However, high uncertainty was associated with the estimates of matrix size and eccentricity (Fig. 4d, e). We did not detect differences in matrix orientation (Fig. 4f), and we did not uncover difference in parts of the trait space through the common-subspace analysis (Fig. 4g).

### Correlations between shape and univariate measurements

**Fig. 5:**
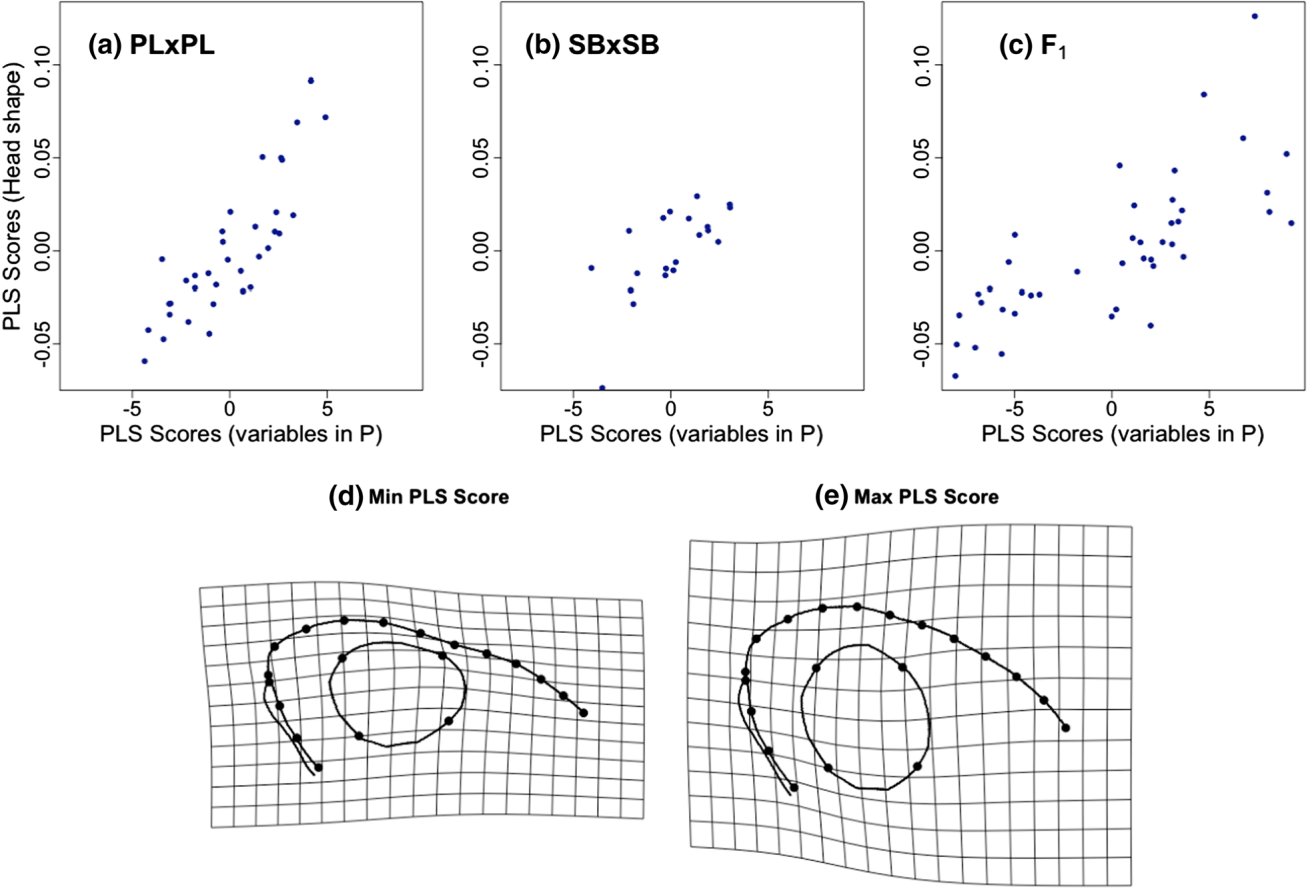
Results of 2-B PLS analysis of head shape and the variables constituting **P**. **a** PL × PL offspring, **b** SB × SB offspring and **c** hybrids. Deformation grids (**d**, **e**) depict the predicted head shapes in the PL × PL offspring at the extreme PLS scores of the block containing the variables of P

We estimated differences among cross types in the propensity of head shape (multivariate data) to covary with the variables in the **P** matrices through two-Blocks Partial Least Squares analyses (2B-PLS) [61]. The 2-B PLS analyses revealed high correlations in all three cross types between head shape and the variables of **P** (PL × PL: PLS_corr_ = 0.84, P < 0.001; SB × SB: PLS_corr_ = 0.70, P = 0.044, F_1_: PLS_corr_ = 0.75; P < 0.001). Together with the shape change grids, the loadings of the first singular vectors indicate that shape changes are mostly associated with the standard length at the onset of exogeneous feeding (Fig. 5, Table 5). However, the strength of the correlation appeared to be lower in SB × SB offspring than the two other cross types, the effect sizes of the PLS analyses being significantly lower in the SB × SB offspring than in the PL × PL offspring and the hybrids (Table 6). Note that removing the SB × SB individual with the lowest head shape PLS score (although not identified as an outlier in the preliminary analyses) made the pairwise differences nonsignificant (results not shown). Cross type-specific wireframes thin-plate spline deformation grids describing shape changes at the extremes the of PLS axis are shown in Additional file 1: Figure S2.

**Table 5:** First singular vector of the first block (variables in P) from each 2-Block Partial Least Square analysis (one per cross type)

	PL × PL	SB × SB	F1
Standard length at D1	− 0.19	− 0.09	− 0.17
Standard length at D3	− 0.94	0.74	− 0.66
Growth D1 to D2	0.00	0.00	0.00
Growth D3 to D4	− 0.02	− 0.01	− 0.01
Yolk sac relative size at D1	− 0.01	− 0.02	− 0.04
Yolk sac conversion rate	− 0.09	0.49	0.73
Feeding latency	0.28	− 0.46	0.05

**Table 6:** Two sample Z-scores between pairs of effect sizes (Z) from the cross type specific 2-Block Partial Least Analyses and associated p-values

Cross types	Z^a^	p-value
PL × PL − SB × SB	2.19	0.03
PL × PL-F_1_	0.08	0.93
SB × SB-F_1_	2.18	0.03

^a^Cross types effect sizes: PL × PL = 3.19; SB × SB = 0.63; F_1_ = 3.85

## Discussion

In our common-garden study, the F_1_ hybrids of two sympatric morphs of Artcic charr showed subtle phenotypic differences with the offspring of the two pure morph crosses. First, while SB and PL charr differed in their growth trajectories (which is in line with previous findings about their life-history strategies, Jonsson et al. [52]), the hybrids differed from the PL charr in their growth (although no difference between the hybrids and the SB charr were observed). However, our results did not provide strong evidence for differences between cross types in average values of yolk sac size and resorption. However, head morphology was dependent on size in the same way for the three cross types (common allometry). The juveniles of the two morphs may therefore differ in shape because of their differences in growth. The PL charr also show higher individual consistency in their propensity to start feeding and tended to be less active and less variable in their feeding behaviour than the SB charr, which is in line with previous observations suggesting that the two morphs have evolved different foraging strategies [57].

The lack of evidence for size-independent head shape variations among cross types contrasts with previous observations of differences between PL and SB embryos in the morphology of craniofacial cartilage elements [55]. These differences might be too subtle to be observed on live specimens in lateral view, and the major morphological differences observed between PL and SB charr might also developed at a later developmental time point than in our study. External, size-independent shape differences have been reported between PL × PL and SB × SB offspring 4 to 6 month after the onset of exogeneous feeding [62]. This age might correspond to a period when their wild conspecifics undergo or have already completed ontogenetic niche shift [63]. Unfortunately, information on the exact timing of the ontogenetic niche shift is lacking, and there are to our knowledge no other appropriate experiments on SB and PL charr at earlier stages to shed light on our results.

Overall, we did not observe evidence of multivariate trait divergence between the two Artic charr morphs. The PL × PL and the SB × SB offspring differed in average value for some traits (especially size and growth), but did not show clearly distinct trait variance–covariance structures. Besides, most of the studied traits appeared uncorrelated. Under multivariate divergent selection, the evolutionary trajectories of populations are expected to be biased in the direction of the phenotypic space with the largest variance (i.e. “lines of least resistance” [64]). These trajectories may be even more complicated by various parameters like the direction of correlational selection relative to the trait with greatest genetic variance, the strength of genetic correlations, the frequency of hybridization and the fitness of hybrids [65, 66]. Genetic covariances and correlations might especially facilitate adaptive changes but also constrain them [67, 68]. In our study, the lack of putative evidence for genetically based trait correlations and the apparent homogeneity of variance among traits (implying the absence of Schluter’s “line of least resistance”) suggest that no evolutionary constraint complicates the divergence of the two morphs. Note, however, that we treated the eggs clutches as a fixed effect when generating the P matrices, because of our limited number of families. Thus, the variance component related to family effects and early environmental variations could not be estimated through variance partitioning. Therefore, our results need to be carefully interpreted considering that these important aspects of phenotypic variation were corrected for but not quantified.

We did not find differences in average trait values that would imply substantial fitness consequences in wild F_1_ hybrids. The hybrids from our study were not strictly intermediate nor transgressive but rather show parental bias (e.g. were similar to the SB × SB offspring in their growth, yolk sac resorption and feeding behaviour). Because the two hybrid cross types were pooled for the analyses, we were not able to test for differences between reciprocal hybrids nor to assess whether one type of hybrids accounted for most of the parental bias. Still, this observation is contrasting with other common-garden experiments reporting intrinsic developmental deficiencies or transgressive characters with obvious ecological implications in F_1_ hybrids between recently diverged populations [37, 69]. For example, hybrids between sympatric charr morphs of Lake Sobachye (Taimyr) develop detrimental ossification anomalies [70], and higher mortalities but intermediate hatching dates were observed in hybrids between lake whitefish ecotypes, *Coregonus cluteaformis* [28]. Considering the parental bias in average trait values observed here and the putative absence of trait correlations, hybrid disadvantages might be occurring (if ever) as functional mismatches. Trait mismatches consist in novel combinations of independent traits with non-intermediate values [71] and may often occur in F_1_ hybrids because of the common effects of dominance [30]. Such functional mismatches also appear plausible in light of the highly numerous regions of differentiation scattered across the entire PL and SB charr genomes [50], suggesting that a diverse suit of traits might have evolved in response to divergent selection.

The lower variance for growth traits observed in the hybrids goes contrary to our predictions of increased phenotypic variability through hybridization. Together with growth in hybrids being as low as in the smallest morph, these observations might be the only hints of developmental deficiencies in the hybrids. Growth-related traits are known for often being highly related to fitness [72, 73], so one may expect slow and lowly variable growth to impact the ecology of hybrids. Of course, consequential developmental unviability as well as novel phenotypes and enhanced phenotypic variability may occur in recombinant (F_2_) and backcrossed hybrids, as observed in many systems [28, 38, 71, 74, 75]. Differences in ecologically relevant traits might also be detectable at later developmental time points than those covered by our study; the ontogenetic niche shift between the two morphs probably occurring as late as several months after the onset of exogeneous feeding [63]. Further studies on later generations of hybrids—although highly constraining regarding the life cycle and the elusive behaviour of the species—may shed more light on the implication of hybridisation regarding postzygotic isolation or phenotypic diversification.

Our results provided little support to the hypothesis of intrinsic postzygotic isolation between the PL and the SB charr (i.e. reproductive barrier produced by environment-independent hybrid deficiencies). Moreover, the singularity of hybrids in terms of average trait values and trait covariance suggests that selection against hybrids might be effective, although these observed differences were subtle, and their fitness consequences are unknown. Thus, the question of reproductive isolation in the two charr remains unresolved. In a recent study on the genetic structure of the two charr, about ten percent of the fishes were identified as potential hybrids [76], so substantial though incomplete reproductive barriers must have evolved between these sympatric morphs and are yet to be discovered. Combined with research on assortative mating and on fine-scale spatiotemporal segregation during spawning, studying the fitness cost of the hybrid characters described above would constitute a promising approach to unravel the evolutionary origins of the Arctic charr morphs of Thinvallavatn.

## Conclusion

Increased trait dimensionality is expected to facilitate local adaptation, sometimes to such an extent that phenotypic divergence can easily occur in the face of high gene flow [66]. Although this should be expected in the SB and the PL charr that seem to be under divergent selection for various trophic and non-trophic traits [77], we did not observe strong evidence for multivariate phenotypic divergence through an extensive phenotypic survey covering different ontogenetic stages. The strongest signal of genetically based differentiation came from growth, which covaried with morphology but not with other traits. Therefore, the divergence of the two morphs might occur without substantial evolutionary constraints nor facilitations. Whether such trend is commonplace or not remains to be established. Northern freshwater fish would be highly suitable model to explore this view. Numerous diverging populations with diverse evolutionary histories, phenotypic distances and reproductive diversification are being extensively studied on the ecological, the genetic and the genomic grounds [42, 43, 78], which now provide consequential resources for multivariate studies on the ontogeny of hybrid phenotypes.

## Methods

### Study system

Thingvallavatn is a deep postglacial lake (surface 84 km^2^, mean depth: 34 m) that formed within a graben of the Mid-Atlantic ridge during the last glacial retreat (*ca.* 10,000 years BP) [79, 80]. The lake is characterized by a wide pelagic zone and three major benthic habitats: a “stony littoral” zone (0–10 m deep) composed of a spatially complex lava substrate with loose stones, crevasses and interstitial spaces, a deeper zone (10–20 m deep), densely vegetated by the algae *Nitella opaca*, and a profundal zone (25 m and deeper) covered by a diatomic gyttja substrate [53, 81]. The lake hosts four morphs of Arctic charr. Two of them, the planktivorous (PL) and the piscivorous charr (PI) feed in the pelagic and epibenthic layers, respectively, and are characterised by a terminal mouth and relatively small pectoral fins [82]. The two other morphs, the large-benthic (LB) and the small-benthic charr (SB), forage in the benthic zone, and show a blunt snout with a subterminal mouth and large pectoral fins [51–53]. The PL and the SB charr are currently found exclusively in sympatry, although coalescent simulations supports evolutionary scenarios involving short periods of geographic isolation [48]. The differentiation of the craniofacial morphology among the two morphs is initiated early during development, before hatching [55], but can also be influenced to some extent by plasticity after the onset of exogeneous feeding [62]. The SB charr spawn from August to November and the PL charr from September to October [58]. The young of the year of the two morphs are believed to use the same habitat, the surf zone (0–1 m deep), from the onset of active feeding in the spring until the summer, when the PL-charr are thought to migrate towards the pelagic and the epibenthic zones [63].

### Fish collection and rearing

We collected mature SB and PL charr with gillnets during five sessions of night fishing in October 2017, at a single spawning site known to be used by both morphs (Svínanesvík, 64° 11′ 24.6ʺ N; 21° 05′ 40.5ʺ W; [58]). We used 52 fish to generate 26 full-sib families on site (crossing design in Additional file 1: Table S4). The eggs were kept at 4.1 ± 0.2 °C in a vertical incubator (MariSource, USA). On the mean hatching day (when 50% of the embryos from a given family had hatched), 40 free-swimming embryos from each one of the first nine families to hatch were moved into single-individual cylinders with a plastic mesh on the lower side to allow water flows (2.2 cm diameter × 6.0 cm height, 0.1 cm^2^ mesh size), and placed into a EWOS tray (60 × 250 cm) with flow-through water. All families and cross types hatched at a similar developmental time point (Additional file 1: Figure S4). Before first feeding (ca. 530 degree days—°C d, March 2018), embryos were moved into 22 cl transparent plastic cups placed in the same EWOS tray (6.1 ± 0.6 °C). These cups were perforated on the sides and were assumed to enable the exchange of olfactory cues and visual contact between congeners. The cups were weekly shuffled inside the setup to overcome eventual confounding effects caused by heterogeneous physical parameters. The fish were fed ad libitum two or three times a day with ground aquaculture pellets (Inicio Plus G 0.4 mm, BIOMAR).

### Data collection

We measured the craniofacial development, pre- and post-feeding growth, and yolk-sac resorption using morphometric data from photographs taken at four points throughout ontogeny: at hatching (ca. 445 °C d), 20 days post-hatching (ca. 530 °C d), 3 to 4 weeks after the onset of exogeneous feeding (ca. 840 °C d) and 9 to 11 weeks after the onset of exogeneous feeding (ca. 1100 °C d). The fish were anaesthetized with 2-phenoxyethanol [83], positioned on their lateral side facing left and photographed with a fixed, down-facing camera (Canon EOS 650D + 100 mm macro lens) before being returned to their respective growing cell. To correct for the tilt caused by the yolk-sac, the specimens were positioned on 3% methyl cellulose [84] for the photographs of the first two timepoints.

The timing of the onset of exogeneous feeding was determined through “One-zero” sampling (i.e. records of the occurrence or non-occurrence of an event within defined observation periods) [59]. Direct observations were made every day on all fish, starting when food was introduced in the rearing setup for the first time (ca. 635 °C d). This was done in the following way: a 3-min observation trial was initiated on each focal individual as the observer introduced food (ca. 10 slowly sinking ground pellets particles of 0.4 mm or less) into the cup of the focal fish. We determined the date of the onset of exogeneous feeding as the date the focal fish was observed catching food for the first time.

Several key aspects of feeding behaviour were estimated by conducting three focal sampling sessions [59] over 3 consecutive days, 7 days after the date of first feeding of the focal individual. We measured behaviours involved in food particle snapping, which constitute a convenient way to study foraging behaviour in captive Arctic charr juveniles [17, 21]. Differences in these behavioural variables were observed between Arctic charr of contrasting sizes (from an aquaculture strain) several weeks after the onset of exogeneous feeding [17]. A 3 min observation period was initiated following the introduction of the food, to record the time it took the fish to seize the first particle (reaction time) [17]. From this point on, an extra 1-min observation trial was initiated, during which feeding intensity (number of particles caught) and feeding strategy (proportion of particles caught on the bottom, on the surface and in mid-water) were recorded. The focal fish was considered “nonfeeding” and the trial was terminated if no particle was seized by the end of the initial 3-min observation period. The observer was not aware of the cross type of the focal individual when conducting the observation trial.

### Digitizing and pre-processing morphological data

Data on size (standard lengths) and morphology were extracted from photographs using Geometric morphometrics methods [85]. We placed landmarks on the tip of the lower jaw, the lower edge of the maxilla below the centre of the eye, the point of maximum curvature between the brain and the cranium, the extremity of the notochord and the anus (Additional file 1: Figure S3). We digitized the contours of the eye, of the head (from the lower edge of the maxilla below the centre of the eye to the point of maximum curvature between the brain and the cranium) and of the yolk sac (from the junction with the vitellin vein to posterior junction with the body) with Bezier curves using the R package Stereomorph. During the standard pre-processing steps (i.e. superimposing the landmark configurations of all specimens to a common coordinate system through Generalized Procrustes Analysis) [86], we estimated the surface of the yolk sac as the area of a polygon composed of 200 semi-landmarks extracted from its respective curve. We calculated the standard length of all specimens as the Euclidian distance between the extremity of the notochord and the furthest of 50 semi-landmarks generated from the curve along the head. The dataset used for the analyses of head shape consisted in 20 landmarks (the 3 initial landmarks located on head, plus 13 and 4 semi-landmarks extracted for the curves around the head and the eye, respectively).

### Analyses of individual traits

We modelled the growth trajectories of every specimen in each cross type using polynomial random regressions [87]. We then tested for overall differences between cross type in the development of the head by conducting phenotypic trajectory analyses of the Procrustes residuals of the head [86]. Morphological disparity analyses [88] were used to compare the types of crosses on the basis of within-group variations in head shapes at the third developmental time-point (3–4 weeks after first feeding). We also tested for group differences in the date of first feeding, feeding intensity, and foraging behaviour with separate GLMMs. The specifications of each model are described in Additional file 1: Table S5. Although reciprocal hybrid crosses were made (numbers in Additional file 1: Table S4), we pooled the hybrids of both maternal origins in the GLMMs to gain sufficient statistical power.

All the GLMMs were run with the R package MCMCglmm [89]. MCMCglmm relies on a Bayesian framework using Markov chain Monte Carlo (MCMC) methods. We always set weakly informative priors (*V* = 1, *nu* = 0.002 or the number of traits for the multi-response models) and determined the optimal number of iterations for model convergence through the examination of trace plots, posterior density plots and effective sample sizes (Additional file 1: Table S5). Inferences were made by comparing the posterior mode estimates and 95% Highest Posterior Density Credible intervals (95% CrI) of each cross type (and in relation to the zero baseline for the significance of *R* estimates).

We studied between-individual variations in feeding behaviour by comparing repeatability estimates among the three cross types. The repeatability of each behavioural variable measured across the three repeated observational trials (propensity to start feeding, number of caught items, vertical location) was calculated according to the formula of adjusted repeatability in [90]. The repeatability estimates of the propensity to start feeding, a variable with binary data, were calculated accounting for Jensen’s inequality when transforming the results (initially on the latent scale) to the data scale, following [91].

### Trait covariance

We studied the patterns of trait covariance by generating a phenotypic matrix of variance–covariance (**P** matrix) for each cross type. **P** matrices are reliable surrogates of genetically based patterns of trait covariances (i.e. of the **G** matrices) when no pedigree is available [64, 92]. **P** matrices are especially likely to be good proxies in our particular study because the effects of the environment were mitigated by the use of common-garden conditions, and because the parental effects were accounted for by including in the subsequent models the family of origin (i.e. the egg clutch) of all individuals. We estimated the components of the three matrices by running three separate Multi-Response Generalized Mixed models [89]. All three models contained seven variables as a response (Table 1). The family was included as a fixed effect while the identity of the individual was included as a random factor. All the traits were mean-standardized by dividing the raw values by their group means [93].

The **P** matrices of each cross type were first compared on the basis of their size, shape and orientation [94]. The matrices sizes (*V*_*tot*_) were used to compare the types of crosses in the overall phenotypic variance and were calculated as the sum of their eigenvalues (Eq. 2 in [95]) [94, 95]. Eccentricity (*Ω*) was used as a measure of the shape of the matrices and was calculated as the ratio of the first two eigenvalues [94]. Differences in overall matrix orientation were assessed using the angles (*θ*) between the first eigenvector of each** P** matrix. Briefly, if the patterns of trait covariances were not conserved but have rapidly evolved among the two morphs, we expected the two types of pure-morph offspring to show differences in the overall size of **P** (*V*_*tot*_), which should suggest a response to two selective regimes eroding genetic variations to different extents. Similarly, differences in eccentricity (*Ω*) between the two purebred offspring were expected (for example, correlational selection, which can produce more constrained, “cigar shaped”, **G** matrices [94], might differ among the respective habitats of each morph). The orientation of **G** can also be subjected to changes because of the effects of correlational selection, among other evolutionary forces [94, 96, 97]. Thus, differences between purebred offspring in the orientation of **P** (*θ*) were also expected [68]. Regarding the hybrids, breakdowns in their trait covariance structure should be indicated by **P** matrices with larger sizes and reduced eccentricity [38]. Meanwhile, differences in the orientation of **P** between the hybrid and the purebred offspring should indicate whether the remaining constraints on the hybrid phenotypes are intermediate, under dominance and conserved relative to one morph, or transgressive (i.e. biased toward a unique direction of the phenotypic space).

Next, we assessed which part of **P** (i.e. which suits of covarying traits) differed the most among cross types in their variance by using Krzanowski’s common subspaces method [60]. This method produces a set of vectors (**H**) that can be used to determine the groups’ similarities in parts of the trait space. Eigenvalues of **H** indicates the degree of resemblance between principal components of the trait subspaces of each group while the eigenvectors are informative of the variables associated with this resemblance. We used the approach of [98], which implements the subspace method in a Bayesian framework. Eigenvalues tending towards the number of measured variables would indicate highly similar subspaces. Significance was assessed through a comparison with eigenvalues generated by randomized **P** matrices (by randomly assigning individuals of each cross types to three groups).

For visualisation purposes, **P** matrices were projected into a subspace composed by the first three eigenvectors **P** matrix of the PL × PL offspring by modifying the plotsubspace() function from [89]. Because angles between eigenvectors are necessarily positive, we compared the angles between the first eigenvectors of **P** with the angles between the first eigenvector of one cross type (depending on the comparison) and the first eigenvector of a “random” **P** matrix. The simulated matrix was generated by sampling 150 individuals from the two cross types being compared.

### Covariance between head shape and univariate traits

Because of the complex multivariate nature of shape data, univariate proxies of shape changes were not used to generate the **P** matrices. Instead, we relied on Two-Blocks Partial Least Squares (2B-PLS) analyses [61] to assess the propensity of head shape at the onset of exogeneous feeding to covary with the variables constituting the **P** matrices. We relied on the method of Adams and Collyer [99] for pairwise comparisons among cross types in the correlation between shape and the other variables. For these analyses only, the latency to first feeding (initially the three measurements per individual) was averaged.

## Supplementary Information

**Additional file 1.** Additional Tables and Figures.

**Additional file 2.** Datsets.

## Data Availability

The datasets supporting the conclusions of this article are included within additional files of the article (Additional file 2). R codes are available at https://github.com/quentin-evo/multi_trait_hybrid_charr/.

## Abbreviations

F_1_: Cross of first generation (here mainly refers to first-generation hybrids)
GLMM: Generalized Linear Mixed-effect Model
2-B PLS: 2-Bocks Partial Least-Square analysis
PL: Planktivorous charr
PL × PL: Cross produced with the gametes of two planktivorous charr
SB: Small benthic charr
SB × SB: Cross produced with the gametes of two small benthic charr
95% CrI: 95% Highest Posterior Density Credible intervals
