# Supplementary material for: Multivariate analysis of morphology, behaviour, growth and developmental timing in hybrids brings new insights into the divergence of sympatric Arctic charr morphs
Source: BMC Ecol Evol. 2021 Sep 7;21:170. doi: 10.1186/s12862-021-01904-8 (PMC8422654; doi:10.1186/s12862-021-01904-8)
Supplement: Supplementary file 1 — Additional file 1. Additional Tables and Figures. [file 12862_2021_1904_MOESM1_ESM.pdf]

Additional file 1: Additional Tables and Figures

Table S1. Pairwise differences of the ontogenetic trajectories of head shape between cross types. Calculated differences, 95% Upper Confidence Limit , standardized scores and *p*-values are shown for three attributes of the trajectories (path length, angle and shape).

	Path length				Angle					Shape			
	Δd	UCLx	Z	p	r	Angle (°)	UCL	Z	p	Δd	UCL	Z	p
**PL-SB**	0.01	0.03	-0.28	0.53	0.99	7.03	9.59	-0.72	0.76	0.14	0.22	-0.34	0.62
**PL-F_1**	0.02	0.04	0.04	0.47	1.00	4.08	6.58	-1.02	0.86	0.09	0.16	-0.66	0.74
**SB-F_1**	0.03	0.05	0.40	0.35	1.00	3.79	6.81	-1.02	0.86	0.15	0.22	-0.33	0.63

Table S2. Posterior estimates of the Linear Mixed-effect Model on the age of exogeneous feeding (degree days). cross SB = SBxSB offspring, cross PL = PLxPL offspring, cross F_1 = hybrids.

	Effect	Posterior mode	95% CrI
**Fixed effects**	Intercept (cross PL)	651.7	642.5 - 662.1
	cross SB	5.1	-10.4 - 17.5
	cross F_1	3.3	-9.1 - 15.0
**Random effect variance**	Family	0.5	0.0 - 146.9
**Residuals**	cross PL	228.3	154.6 - 377.6
	cross SB	323.1	226.1 - 626.9
	cross F_1	253.5	200.7 - 387.4

Table S3. Latency of the focal individual to start feeding across the three observation trials (log seconds).

	Posterior mode	95% CrI
**Trial 1**	3.56	2.60 - 4.25
**Trial 2**	3.32	2.37 - 4.01
**Trial 3**	3.55	2.51 - 4.21
**Cross SBxSB**	-0.33	-1.54 - 0.76
**Cross F_1**	-0.17	-1.27 - 0.68
**Trial 2*Cross SBxSB**	0.53	-0.53 - 1.70
**Trial 3*Cross SBxSB**	0.20	-0.66 - 1.55
**Trial 2*Cross F_1**	0.03	-0.89 - 0.90
**Trial 3*Cross F_1**	0.08	-0.70 - 1.14

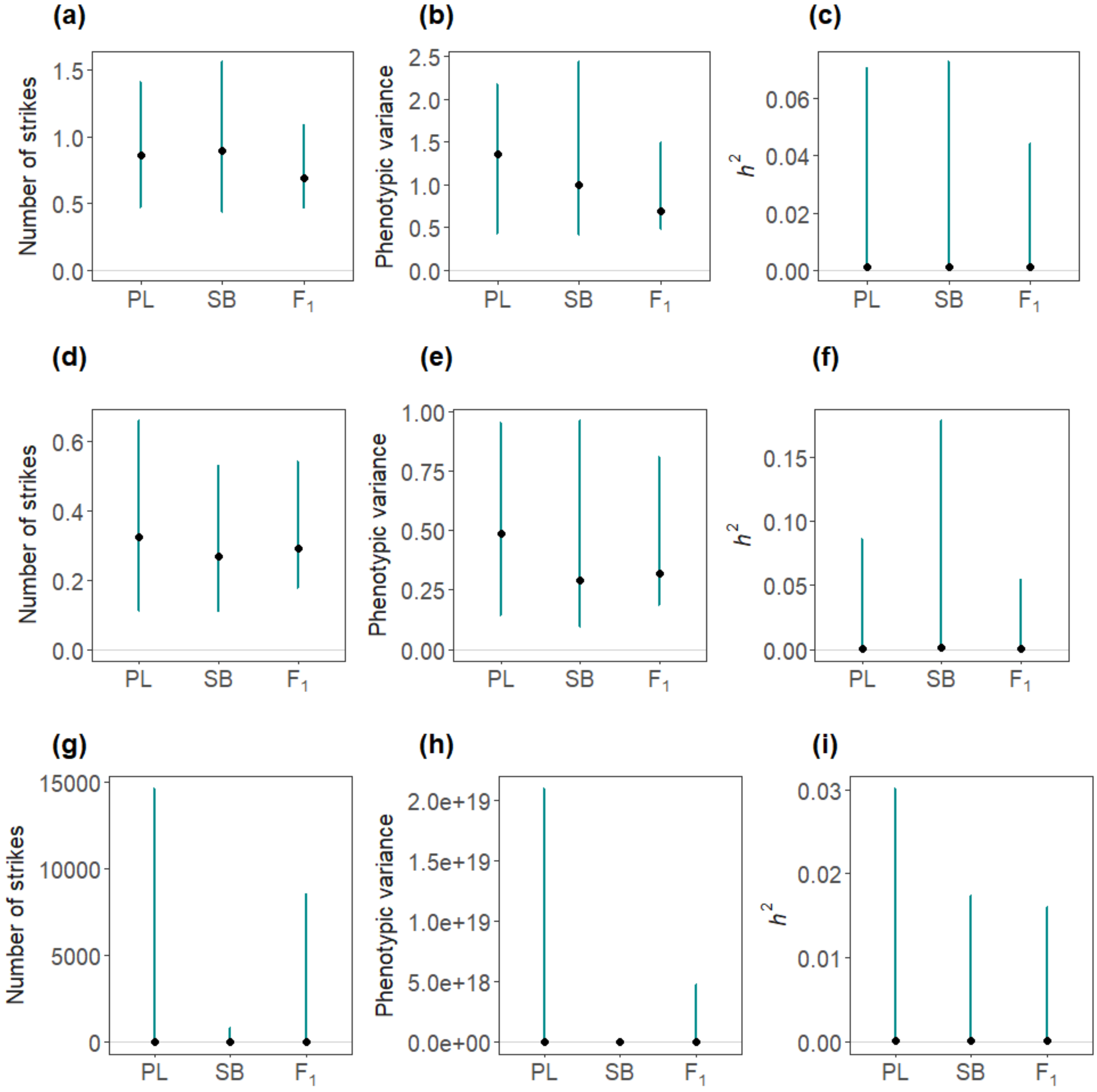

Fig S1. Estimates of feeding behaviours. (a-c) Numbers of feeding attempts on the bottom of the cup, (d-f) at mid-water, (g-i) at the surface. (a-g) Fixed effect estimate, (d-h) total within group variance, (c-i) heritability (h^2). Categories: SB = SBxSB offspring, PL = PLxPL offspring, F_1 = hybrids. Dots: Posterior modes, bars: 95% Credible Intervals.

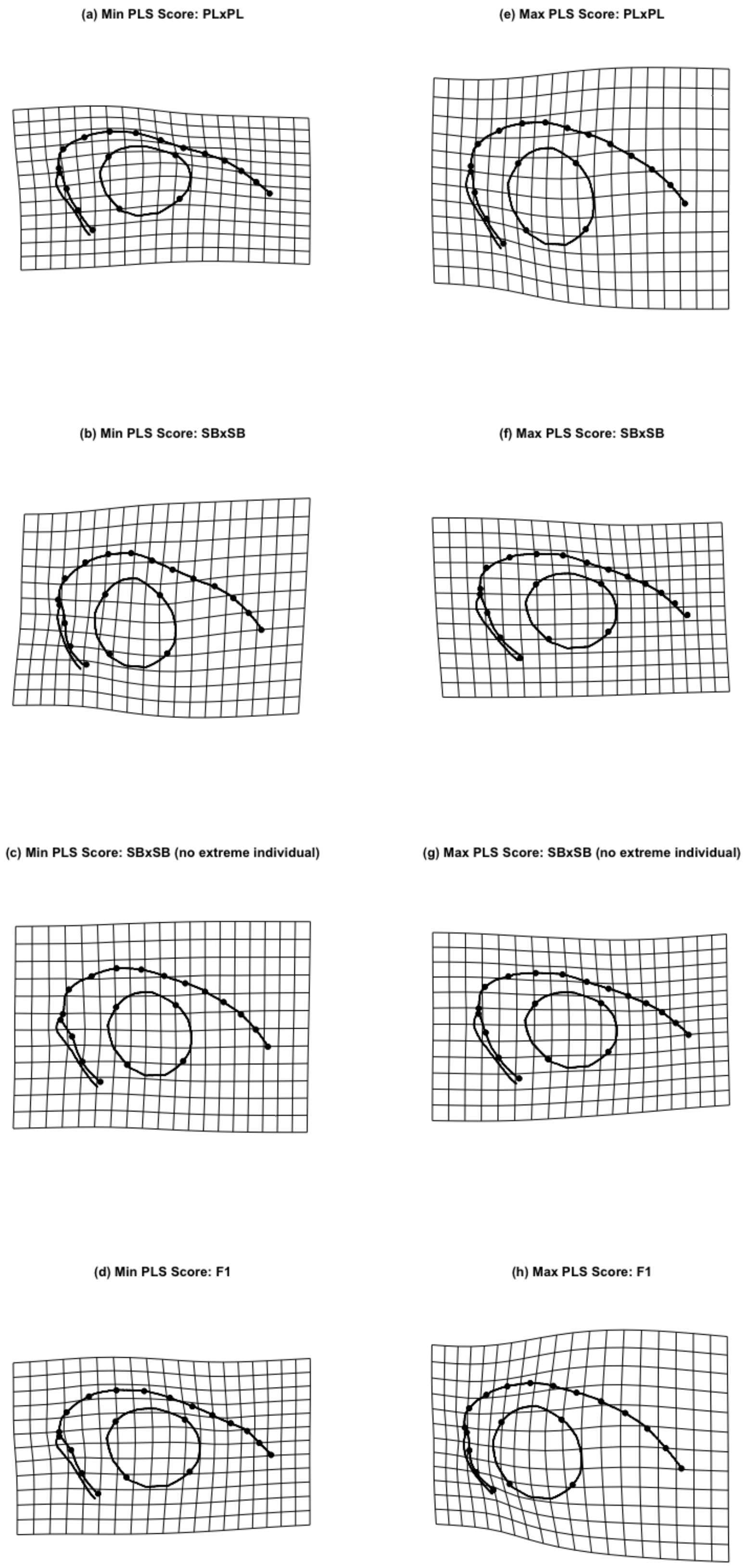

Fig. S2. Thin-plate spline deformation grid depicting shape changes extreme of the PLS axis. (a,e) pure PL offspring, (b-f) pure SB offspring, (d-h) F_1 hybrids.

Table S4. Crossing design of the rearing experiment. Cross types: Female gamete x Male gamete. Parents were used for crosses only once (no split families). The numbers of individuals on the left refer to the maximum number individuals at the start of the experiment. The numbers on the right refer to the number of individuals available with no missing data among all the sampling steps. One SBxPL family (19 individuals) hatched two weeks after the others and was not used for the analyses on trait covariance, growth, morphology and feeding behaviour.

Cross type	Number of families	Number of individuals
PLxPL	2	64 - 37
SBxSB	2	42 -15
PLxSB	3	75 - 23
SBxPL	2	49 -18

(a)

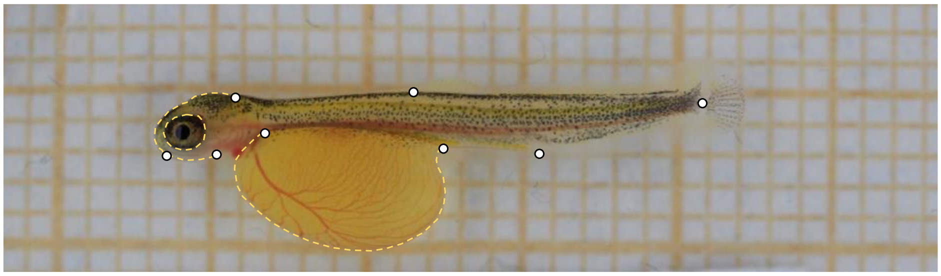

(b)

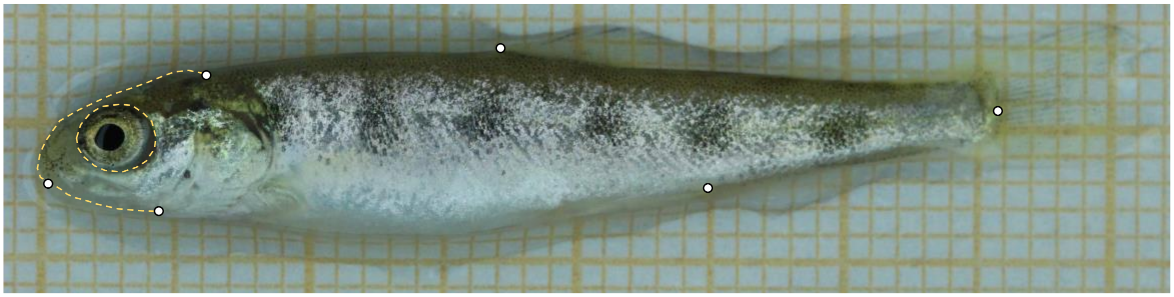

Fig. S3 Landmarks used for the analyses of shape differences. (a) On free-swimming embryos at hatching (D1: ca. 445 °C *d*ays) and (b) on actively feeding juveniles (D4: ca. 1100 °C *d*ays). The dashed curves depict the location of the Bezier curves used to extract the semi-landmarks. The specimens presented here are from the PLxPL offspring.

Table S5. Specifications of the Generalized linear mixed-effect models run the analyses to the separate traits. For all model, burnin = 300 x number of iterations, thinning intervals = 10 x number of iterations.

Trait	Fixed effects	Random variable	Model specificities	Number of iterations
**Growth**				
Log standard length (average differences)	Cross type x Age	Family Individual	Random regression Second order polynomial regression	$6.5x10^4$
Log standard length (within-group variation)	Age + Family	Individual	One model per type of cross	$1.3x10^6$
**Yolk sac size at hatching and resorption**				
Yolk sac area at hatching, Yolk sac area at hatching + 20 days (average differences)	Standard length + Cross type	Family Individual	Multi-response model	$3.9x10^6$
Yolk sac area at hatching, Yolk sac area at hatching + 20 days (within-group variation)	Standard length Family	Individual	Multi-response model One model per type of cross	$4.6 x10^6$
**Feeding behaviour**				
Age of exogeneous feeding	Cross type	Family	Binomial GLMM with logit link	$2.6 x10^5$
Propensity to feed	Feeding trial x Cross type	Family Individual		$3.9 x10^6$
Log Latency to feed	Feeding trial x Cross type	Family Individual		$2.6 x10^5$
Total Number of feeding attempts	Feeding trial x Cross type	Family Individual	GLMM with log link	$5.7 x10^7$
Number of feeding attempts (bottom)	Total Number of feeding attempts + Cross type	Family Individual	GLMM with log link	$1.0 x10^7$
Number of feeding attempts (water column)	Total Number of feeding attempts + Cross type	Family Individual	GLMM with log link	$1.0 x10^7$
Number of feeding attempts (surface)	Total Number of feeding attempts + Cross type	Family Individual	GLMM with log link	$1.0 x10^7$

Table S6. List of the individuals that were discarded from the analyses.

	Type of cross	Explanation
**Removed from the morphological analyses at hatching**	PLxPL	Identified as outlier in MANOVAs on body shape at hatching. Malformed mandibula and odd yolk sac shape on the photographs.
	SBxSB	Heavily malformed craniofacial morphology ("bulldog" face)
	SBxSB	Heavily malformed craniofacial morphology ("bulldog" face)
**Removed from the behavioural analyses**	PLxSB	Individual unable to adjust its buoyancy
	SBxPL	Twisted spine constraining swimming activities
	PLxSB	Head infected by fungi
	SBxSB	Twisted spine constraining swimming activities
	SBxSB	Individual unable to adjust its buoyancy
	PLxPL	Yolk sac not depleted and containing air

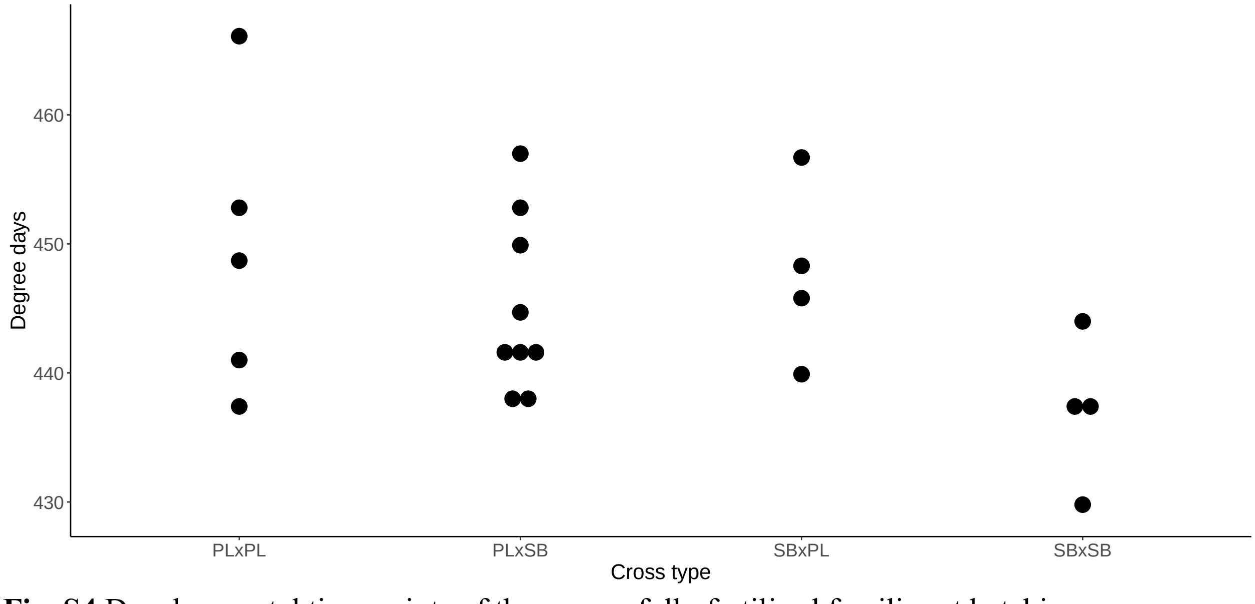

Fig. S4 Developmental time points of the successfully fertilised families at hatching.
